# Supplementary material for: Bio-Compatibility and Bio-Insulation of Implantable Electrode Prosthesis Ameliorated by A-174 Silane Primed Parylene-C Deposited Embedment
Source: Micromachines (Basel). 2020 Nov 30;11(12):1064. doi: 10.3390/mi11121064 (PMC7761135; doi:10.3390/mi11121064)
Supplement: Supplementary file 1 [file micromachines-11-01064-s001.pdf]

SUPPLEMENTARY INFORMATION

Bio-compatibility and Bio-insulation of Implantable Electrode Prosthesis Ameliorated by A-174 Silane Primed Parylene-C Deposited Embedment

Chin-Yu Lin[1,2], *Wan-Shiun Lou*[3], *Jyh-Chern Chen*[4], *Kuo-Yao Weng*[5], *Ming-Cheng Shih*[3], *Ya-Wen Hung*[3], *Zhu-Yin Chen*[1] *and Mei-Chih Wang* [3]*

[1]Institute of New Drug Development, China Medical University, No.91 Hsueh-Shih Road, Taichung, 40402, Taiwan.

[2] Master Program for Biomedical Engineering, Collage of Biomedical Engineering, China Medical University, No.91 Hsueh-Shih Road, Taichung, 40402, Taiwan.

[3]Biomedical Technology & Device Research Laboratories, Industrial Technology Research Institute, No.195, Sec.4, Chung Hsing Rd., Chutung, Hsinchu, 31057, Taiwan.

[4]ACE Biotek Co., Ltd., No.66, Shengyi 2nd Rd., Jhubei, Hsinchu, 30261, Taiwan.

[5]Technology Transfer and Law Center, Industrial Technology Research Institute, No.195, Sec.4, Chung Hsing Rd., Chutung, Hsinchu, 31057, Taiwan.

*To whom correspondence should be addressed: Chin-Yu Lin (geant@mail.cmu.edu.tw) and Mei-Chih Wang (mcwang@itri.org.tw)

Assistant Professor Dr. Chin-Yu Lin, Institute of New Drug Development, College of Medicine, China Medical University, No.91 Hsueh-Shih Road, Taichung, 40402, Taiwan. Tel: +886-4-22053366 # 8108; Fax: +886-4-22051507.

Dr. Mei-Chih Wang, Biomedical Technology & Device Research Laboratories, Industrial Technology Research Institute, No.195, Sec.4, Chung Hsing Rd., Chutung, Hsinchu, 31057, Taiwan. Tel: +886-3-5912812; Fax: +886-3-5820369.

Supplementary Methods

Cytotoxicity evaluation of bio-compatibility test

According to ISO 10993-5 and ASTM F813 - 07(2012), the L929 mouse fibroblasts were treated with eluted concentrates extracted from parylene-C and polyimide embedded IDE chips, respectively. Briefly, L929 cells were seeded 1 $\times 10^5$/per well in 6-well plate overnight in 37 °C, 5% CO_2 incubator. The 0.6 g-weighed encapsulated IDE chips were immersed in 3 ml MEM complete medium containing 10% FBS for 72 h at 37 °C and the eluted extract was collected. The concentrates eluted from identical weight of high-density polyethylene (HDPE) tube and latex rubber were served as negative and positive control, respectively. The culture medium MEM containing 10% FBS / 1% antibiotics was served as reagent Control. The eluted concentrate was added into L929 plate and the cytotoxicity was measured at 24 h post-incubation in

compliance with the manufacturer's instruction (Crystal violet Assay Kit, Cat. ab232855, Abcam Inc., US). The cytotoxicity was qualitatively graded from 0-4 according to the morphological condition (Table S1).

For cell viability assay, L929 cells in 96 well plate was treated with eluted concentrate contained MEM complete medium for 24h, cell mediums were examined by MTS Cell Proliferation Colorimetric Assay Kit (Biovision, Cat. K300) and detected absorbance at O.D. 490 nm in compliance with manufacturer's instruction.

Table S1. Qualitative morphological grading of cytotoxicity

Grade	Reactivity	Conditions and responses of all cell cultures
0	None	Discrete intracytoplasmatic granules, no cell lysis, no reduction of cell growth
1	Slight	Not more than 20% of the cells are round, loosely attached and without intracytoplasmatic granules, or show changes in morphology; occasional lysed cells are present; only slight growth inhibition observable.
2	Mild	Not more than 50 % of the cells are round, devoid of intracytoplasmatic granules, no extensive cell lysis, not more than 50 % growth inhibition observable.
3	Moderate	Not more than 70 % of the cell layers contain rounded cells or are lysed; cell layers not completely destroyed, but more than 50 % growth inhibition observable.
4	Severe	Nearly complete or complete destruction of the cell layers.

Hemolysis evaluation of bio-compatibility test

According to ISO10993-4 and ASTM F756-08 guidance to measure the hemolytic safety concern raised from implantable medical devices. Rabbit blood cells could be served as an excellent cell source for hemolytic assessment due to their high sensitivity. Blood collected from three rabbits was mixed and examined the value of plasma free hemoglobin. The blood containing < 2 mg/ml free hemoglobin could be selected for subsequent hemolytic assessment in compliance with manufacturer's instruction (Drabkin's reagent, Cat. D5941, Sigma-Aldrich). To prepare the eluted concentrates, parylene C or polyimide coated SiO_2 wafer (30 cm^2 each) was immersed in 10 ml PBS (Ca^{2+} and Mg^{2+} free) for 72 h at 37 °C, and identical-sized HDPE was served as negative control with identical elution process. For blank and positive control, 10 ml PBS and sterile water without any material were prepared. All testing solutions were withdrawn 7 ml to mix with 1 ml whole blood for 3 h at 37 °C with gentle mixing, centrifuged at 800G for 15 min, and withdrew 1 ml supernatants to mix with 1 ml Drabkin's solution for 15 min at room temperature, then measured the absorbance at 540 nm wavelength. The blank corrected hemolysis (%) was calculated as follow: (A^S: absorbance of supernatant from testing material or control, A^T: absorbance from total blood hemoglobin, A^B: absorbance from blank)

$$Blank\ corrected\ hemolysis\ (\%) = \frac{A^S - A^B}{(0.844)A^T - A^B} \times 100\%$$

The hemolytic index = blank corrected hemolysis (%) of testing sample- negative control, the hemolytic index value between 0-2 could be classified as non-hemolysis (Table S2).

Table S2. Hemolytic assessment according to the hemolysis index

Hemolytic index above the negative control	Hemolytic grade
0-2	Non-hemolysis
2-5	Slightly hemolysis
>5	Hemolysis

Irritation evaluation of bio-compatibility test

Table S3. Scoring criteria for irritation evaluation.

Score	Erythema (ER)	Edema (ED)
0	No erythema	No edema

1	Very slight erythema (barely perceptible)	Very slight edema (barely perceptible)
2	Well-defined erythema	Well-defined edema (edges of area well-defined by defined raising)
3	Moderate erythema	Moderate edema (raised approximately 1 mm)
4	Severe erythema (beet redness) to eschar formation	Server edema (raised more than 1 mm, and extending beyond exposure area)

Hypersensitivity evaluation of implantation test

Table S4. Scoring criteria of cell types and responses in the histopathological evaluation.

Cell types / responses	Score				
	0	1	2	3	4
Polymorphonuclear cells	0	Rare, 1-5 / phf[a]	5-10 / phf	Heavy infiltrate	Packed
Lymphocytes	0	Rare, 1-5 / phf	5-10 / phf	Heavy infiltrate	Packed
Plasma cells	0	Rare, 1-5 / phf	5-10 / phf	Heavy infiltrate	Packed
Macrophages	0	Rare, 1-5 / phf	5-10 / phf	Heavy infiltrate	Packed
Giant cells	0	Rare, 1-2 / phf	3-5 / phf	Heavy infiltrate	Packed
Necrosis	0	minimal	mild	moderate	Severe

[a]: phf= per high powered (400×) field.

Table S5. Scoring criteria of neovascularization, fibrosis and fat infiltrate in the histopathological evaluation.

Response	Score				
	0	1	2	3	4
Neovascularization	0	Minimal capillary proliferation, focal, 1-3 buds	Groups of 4-7 capillaries with supporting fibroblastic structures	Broad band of capillaries with supporting structures	Extensive band of capillaries with supporting fibroblastic structures
Fibrosis	0	Narrow band	Moderately thick band	Thick band	Extensive band
Fatty infiltrate	0	Minimal amount of fat associated with fibrosis	Severe layers of fat and fibrosis	Elongated and broad accumulation of fat cells around the implant site	Extensive fat completely surrounding the implant

Table S6. Overall irritant rating of subcutaneous implantation analysis determined by total scores.

Scores	Irritant status
0.0-2.9	Non-irritant
3.0-8.9	Slight irritant
9.0-15.0	Moderate irritant
>15	Severe irritant

Supplementary Figures

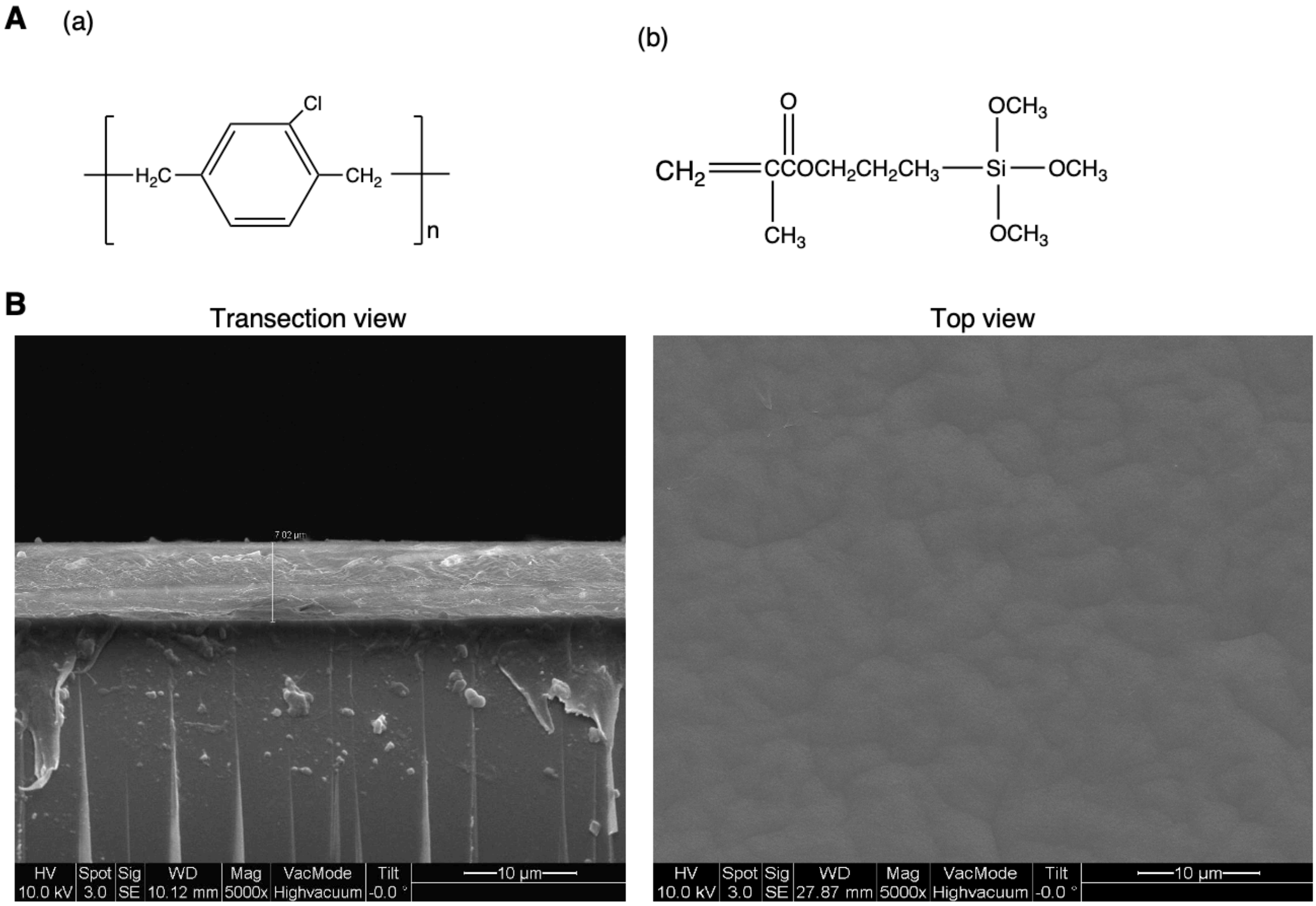

Fig. S1. Encapsulation of IDE chip with parylene-C and A-174, examined by SEM. (A, a) Molecular structure of parylene-C. (A, b) Molecular structure of gamma-methacryloxypropyltrimethoxysilane (Silquest A-174 silane). (B) Representative SEM images of IDE chips embedded with parylene C.

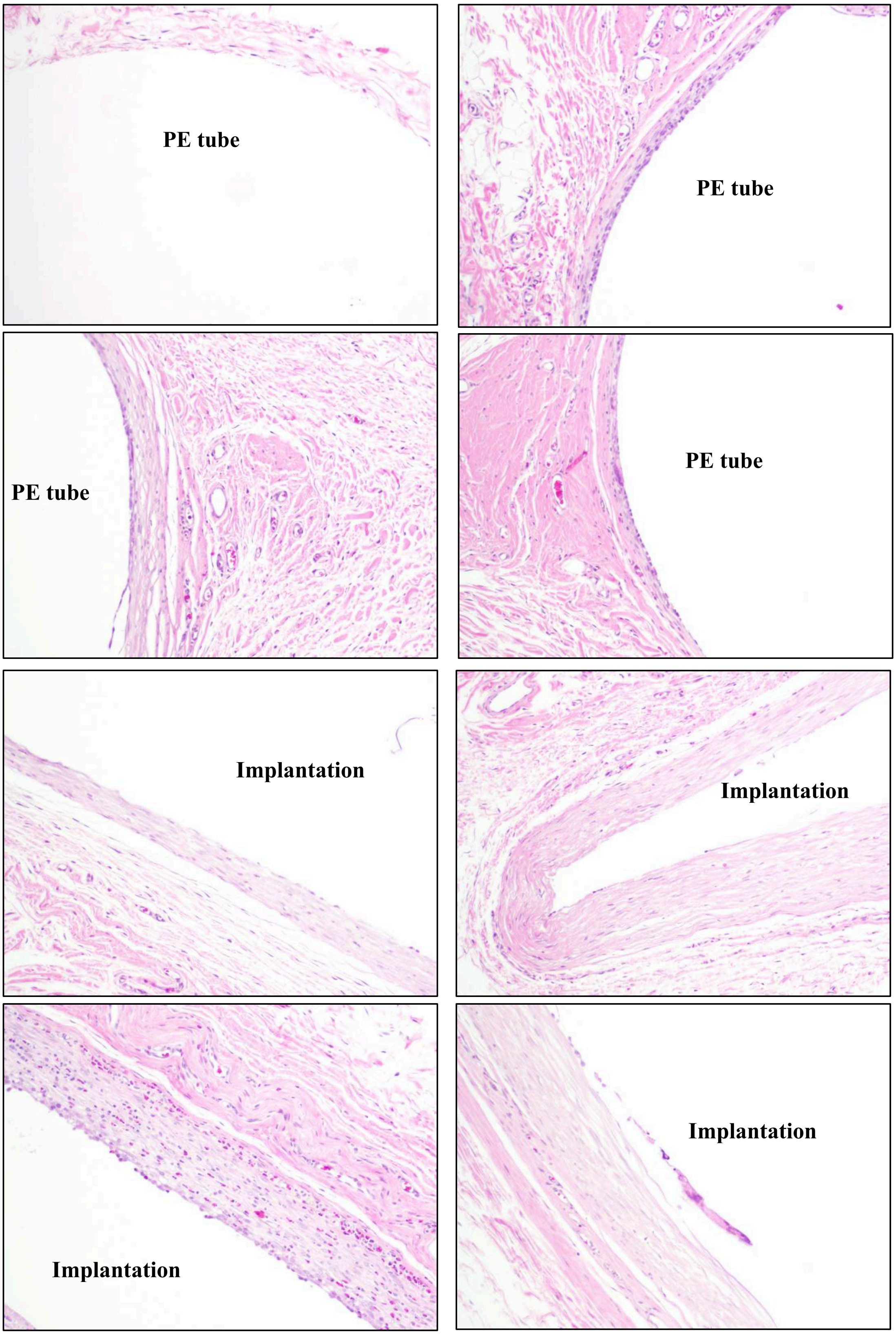

Fig. S2. ISO 10993-6 Hypersensitivity examination of electrode prosthesis through subcutaneous implantation. H&E staining of subcutaneous tissues surrounding the graft at 12 weeks post-implantation. Animal #2 (N=3). White area indicates the implantation site, "PE tube" indicates HDPE implantation, "Implantation" indicates IDE chip implantation. (Magnification: 100×)

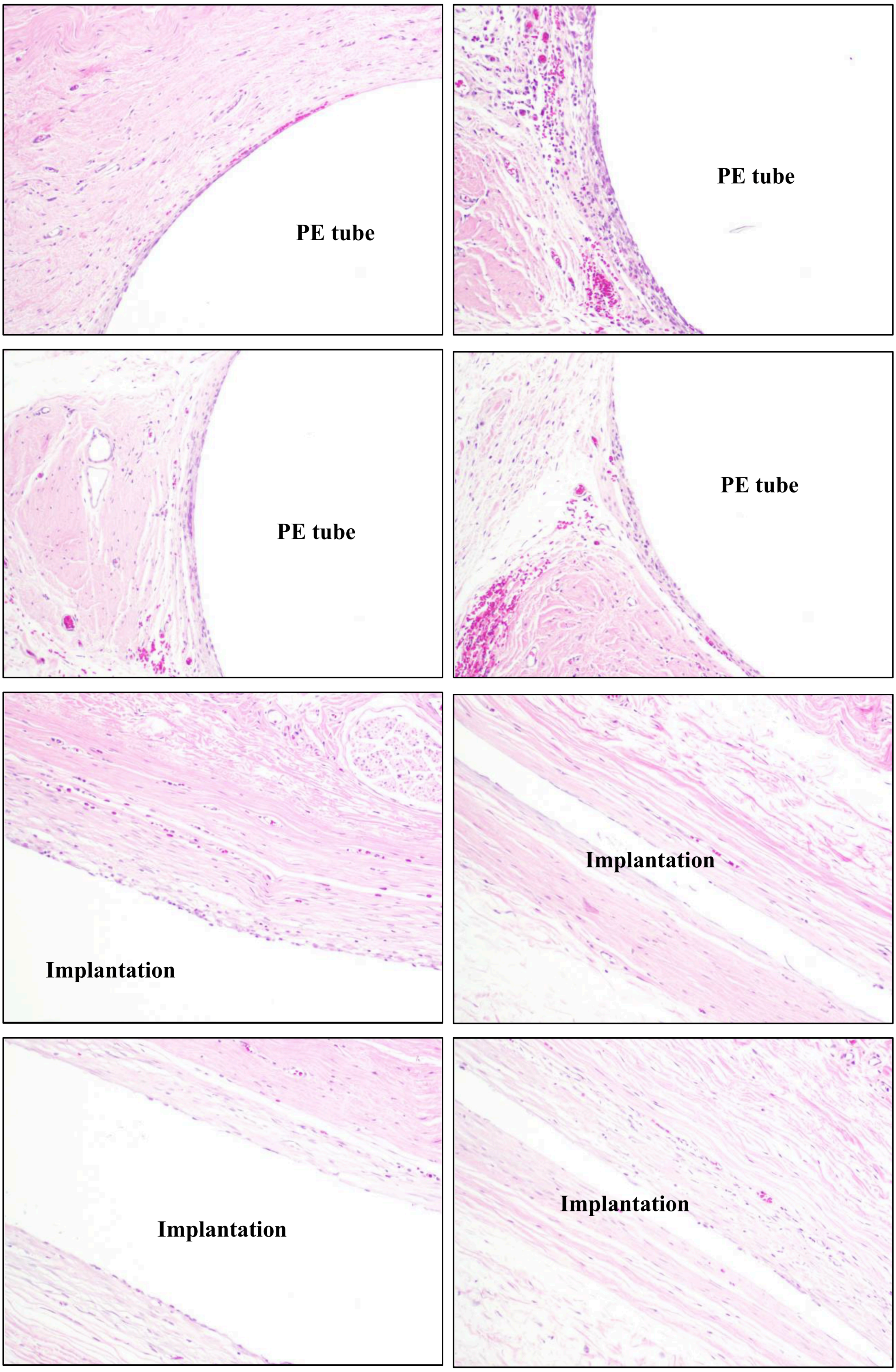

Fig. S3. ISO 10993-6 Hypersensitivity examination of electrode prosthesis through subcutaneous implantation. H&E staining of subcutaneous tissues surrounding the graft at 12 weeks post-implantation. Animal #3 (N=3). White area indicates the implantation site, “PE tube” indicates HDPE implantation, “Implantation” indicates IDE chip implantation. (Magnification: 100×)
